# Long-term prognosis after kidney donation: a propensity score matched comparison of living donors and non-donors from two population cohorts

**DOI:** 10.1007/s10654-020-00647-y

**Published:** 2020-05-21

**Authors:** Shiromani Janki, Abbas Dehghan, Jacqueline van de Wetering, Ewout W. Steyerberg, Karel W. J. Klop, Hendrikus J. A. N. Kimenai, Dimitris Rizopoulos, Ewout J. Hoorn, Sylvia Stracke, Willem Weimar, Henry Völzke, Albert Hofman, Jan N. M. Ijzermans

**Affiliations:** 1 Department of Surgery, Division of HPB and Transplant Surgery, Erasmus University Medical Center, Rotterdam, The Netherlands; 2 Department of Epidemiology, Erasmus University Medical Center, Rotterdam, The Netherlands; 3 Department of Epidemiology and Biostatistics, School of Public Health, Imperial College London, London, UK; 4 Department of Internal Medicine, Division of Nephrology and Transplantation, Erasmus University Medical Center, Rotterdam, The Netherlands; 5 Department of Public Health, Erasmus MC University Medical Center, Rotterdam, The Netherlands; 6 Department of Biomedical Data Sciences, Leiden University Medical Center, Leiden, The Netherlands; 7 Department of Biostatistics, Erasmus University Medical Center, Rotterdam, The Netherlands; 8 Institute for Community Medicine, Ernst Moritz Arndt University Greifswald, Greifswald, Germany; 9 Department of Epidemiology, Harvard T.H. Chan School of Public Health, Boston, MA USA

**Keywords:** Live kidney donor, Renal function, Long-term, Outcome

## Abstract

**Background:**

Live donor nephrectomy is a safe procedure. However, long-term donor prognosis is debated, necessitating high-quality studies.

**Methods:**

A follow-up study of 761 living kidney donors was conducted, who visited the outpatient clinic and were propensity score matched and compared to 1522 non-donors from population-based cohort studies. Primary outcome was kidney function. Secondary outcomes were BMI (kg/m^2^), incidences of hypertension, diabetes, cardiovascular events, cardiovascular and overall mortality, and quality of life.

**Results:**

Median follow-up after donation was 8.0 years. Donors had an increase in serum creatinine of 26 μmol/l (95% CI 24–28), a decrease in eGFR of 27 ml/min/1.73 m^2^ (95% CI − 29 to − 26), and an eGFR decline of 32% (95% CI 30–33) as compared to non-donors. There was no difference in outcomes between the groups for ESRD, microalbuminuria, BMI, incidence of diabetes or cardiovascular events, and mortality. A lower risk of new-onset hypertension (OR 0.45, 95% CI 0.33–0.62) was found among donors. The EQ-5D health-related scores were higher among donors, whereas the SF-12 physical and mental component scores were lower.

**Conclusion:**

Loss of kidney mass after live donation does not translate into negative long-term outcomes in terms of morbidity and mortality compared to non-donors.

**Trial registration:**

Dutch Trial Register NTR3795.

**Electronic supplementary material:**

The online version of this article (10.1007/s10654-020-00647-y) contains supplementary material, which is available to authorized users.

## Introduction

Each year, nearly 5000 healthy individuals in Europe and 6000 in the United States donate a kidney to help patients with end-stage renal disease (ESRD) [1, 2]. Potential living donors are extensively screened by transplant professionals, who select only those donors whose health is unlikely to be compromised by donation. The surgical donation procedure has been demonstrated to be safe with a low risk of peri-operative morbidity and a very low risk of procedure-related mortality [3, 4]. However, donors should be aware of the implications of donation in their future life. Compared to pre-donation levels, eGFR initially decreases after donation [5] but seems to remain stable with no further decrease after more than a decade [6, 7].

Recently, unfavorable results from donor versus non-donors studies have emerged. Previously, it was assumed that donors have no increased risk of mortality [4, 8–10], ESRD [8, 11–13], or gestational hypertension [8, 14] compared to non-donors. However, recent single-center and national registry studies on long-term follow-up outcomes comparing donors to non-donors have reported an increased risk of mortality [15], ESRD [15–17], gestational hypertension, and pre-eclampsia [18] among donors.

Determining the long-term impact of living donation is essential and criteria are needed to identify the donors at risk in the long-term. Current literature presents conflicting results regarding safety of live kidney donors at follow-up, in which the flaw of comparability of studied donors and matched non-donors is key to these conflicting results [19]. Therefore a more accurate selection of non-donors is necessary. Furthermore, low absolute risk among donors creates uncertainty in estimates when adjusting for potential confounders [19]. It is important that comprehensive data become available for this process to strive for a similar health status between donors and non-donors, allowing proper analysis of the donation procedure with regard to risk factors. The safety of these healthy individuals who are not patients themselves is paramount and demand more data to validate live kidney donation on the long-term. In the present study, we aim to overcome limitations of previous studies by comparing donors to non-donors using propensity score matching to evaluate the long-term effects on serum creatinine and eGFR as well as outcomes, including hypertension, diabetes mellitus, cardiovascular events, survival, and quality of life.

## Materials and methods

### Study design

The conduct and reporting of the study followed STROBE guidelines for observational studies [20] (Supplemental Table S1). The study was designed by the authors [21] and approval obtained by the medical ethics committee of Erasmus University Medical Center, Rotterdam, the Netherlands (MEC-2012-519). Informed consent was obtained from all study participants. We initiated a propensity-score matched follow-up study using individual level donor data from Erasmus University Medical Center, and comparison data from two population-based follow-up studies. All eligible donors were invited for an extensive study visit that included self-reported medical history, and an interview-based questionnaire on quality of life, supplementary to their annual physical examination and laboratory tests. The study visits were conducted between August 19, 2013, and December 31, 2014. To increase the response among donors, the visits were also conducted at Radboud University Medical Center in Nijmegen and Admiraal de Ruyter Hospital in Goes in different provinces of the Netherlands. Donors were included in the study if they were still alive during the inclusion period, lived in the Netherlands, and visited one of the study hospitals or filled out self-report forms. All questionnaire-related interviews were conducted by one of the three investigators. Subjects who declined to participate were asked to fill out self-report forms on their medical history and quality of life and permission was requested for access to their medical records in order to analyze potential selection bias.

### Data sources

All data were obtained from self-reporting and interview-based questionnaires, physical examination, and laboratory tests. To ensure the accuracy and completeness of the donor data, we manually reviewed the pre-donation and annual follow-up medical records in the hospital’s electronic patient database. We also obtained information from three linked databases containing the municipal administration records on vital status and demographic characteristics for all inhabitants, and the registry for dialysis patients. Outcome data were complete for all variables in this study except for quality of life scores among non-donors.

### Population

#### Donors

We included all individuals who donated a kidney from 1981 through 2010 at the Department of Surgery of Erasmus University Medical Center or who had their full medical work-up performed there prior to donation but donated at another transplant center because of their participation in the national kidney exchange transplant program. [22] We identified 1092 donors eligible for this study (See Supplemental Figure S1 for an overview of the number of donations per year).

#### Non-donors

Inhabitants of Western Europe have similar donation and transplantation legislation, lifestyles, and healthcare systems and access. [23–25] Therefore, non-donors were selected from the Study of Health in Pomerania (SHIP) [26] in Germany, a population-based cohort study with participants aged 20–70 years, and the Rotterdam Study [27] in the Netherlands, a population-based cohort study with participants aged 45 years and older. SHIP is a population-based cohort study initiated in 1997 among inhabitants of West Pomerania in the north-east of Germany. Two main objectives of this study were first to assess prevalence and incidence of common risk factors, subclinical disorders and clinical diseases, and second to investigate the complex associations among these. The Rotterdam Study is a prospective cohort study that started in 1990 in Ommoord, a district of the city of Rotterdam, the Netherlands. The study analyzes determinants of cardiovascular, endocrine, hepatic, neurological, ophthalmic, psychiatric and respiratory diseases. Both population-based studies were selected to cover the whole age range of our donors. Participants from the SHIP-0 cohort who enrolled between 1997 and 2001 (n = 4308) were selected based on sufficient follow-up time. Participants from the Rotterdam Study II and III cohorts who enrolled between 2000 and 2001 or 2006 and 2008 respectively (n = 6943), were selected since cohort I did not included all studied outcomes at baseline. Data were taken from the latest follow-up examinations of both cohorts, 2012 for the SHIP and 2015 for the Rotterdam Study.

### Study outcomes

All study subjects were followed until death, emigration out of the country, or the end of the examination period (April 20, 2016, for donors, and December 31, 2015, for non-donors). The primary outcome was serum creatinine (μmol/l) and eGFR (ml/min/1.73 m^2^; calculated using the CKD-EPI formula [28]), which was measured at baseline, one year after donation (donors only), and at long-term follow-up. ESRD was defined as necessitating dialysis or transplantation. Secondary outcomes were incidence of hypertension (defined as use of antihypertensive medication, systolic blood pressure ≥ 140 mmHg, or diastolic blood pressure ≥ 90 mmHg), incidence of diabetes (defined as use of antidiabetic medication or glucose ≥ 7 mmol/l with diet), BMI (kg m^2^), incidence of cardiovascular events (defined as myocardial infarction, percutaneous coronary intervention, coronary artery bypass surgery, or cerebral vascular accident), cardiovascular mortality (defined as death by cardiovascular event), mortality (censor date April 20, 2016, for donors; December 31, 2012 for the SHIP; and December 31, 2015, for the Rotterdam Study), and quality of life measured by ShortForm-12 (SF-12) [29] and EuroQoL (EQ-5D) [30]. Both quality of life questionnaires were used since the Rotterdam Study only included the EuroQoL and SHIP the ShortForm-12. The Short Form health questionnaire is a validated and commonly used tool to measure health related quality of life ranging from a score between 0 and 100. It contains questions on physical performance and well-being, and mental functioning and emotional well-being, resulting in the physical (PCS) and mental component score (MCS) respectively. The EQ-5D records quality of life in five dimensions: mobility, self-care, daily activities, pain or discomfort, and anxiety/depression. The responses on the five dimensions combine to a score according to Dutch tariff [31] between − 0.59 (worst imaginable health state) and 1.00 (best imaginable health state).

### Propensity score matching

Restriction, multiple imputation, and matching were used to select a cohort of non-donors that was just as healthy as the donors (Supplemental Methods). We restricted the sample of all Rotterdam Study and SHIP participants (n = 11,251) to eligible non-donors without identified contraindications for donation (n = 9270) at the time of enrollment in the population-based cohort studies, including pre-existing diabetes, an estimated glomerular filtration rate (eGFR) < 60 ml/min/1.73 m^2^ (defined by blood or urine analysis), and BMI > 40 kg/m^2^. To account for the fact that the data on covariates at baseline were not complete for all subjects, a multiple imputation approach was utilized to impute missing covariate values based on the method of chained equations [32]. Using this procedure, 20 complete data sets were created. For each imputed data set, non-donors were matched to donors with replacement using propensity score matching [33, 34]. Balance between the donor and non-donor groups was checked with summary measures of Q–Q plots comparing the covariates in the matched groups [35]. Initially, a 4:1 match was targeted [21], but the ratio was reduced to 2:1 to allow for optimal balance. Matching was based on baseline characteristics of age (years), gender, year of donation/enrollment in the population-based cohort study, weight (kg), height (cm), ethnicity, eGFR (ml/min/1.73 m^2^), systolic and diastolic blood pressure (mmHg), pre-existing hypertension, pre-existing cardiovascular events, serum glucose level (mmol/l), current smoking, alcohol use, and education level.

### Statistical analysis

Baseline characteristics were compared using the Kruskal–Wallis test for continuous variables and the Fisher’s exact test for dichotomous variables, without accounting for matching. All analyses were performed for each of the complete data sets and the results from the analysis of each imputed data combined using Rubin’s formulas [36]. The analyses of continuous outcomes were based on linear regression, the analyses of dichotomous outcomes on logistic regression, and the analysis of survival on Cox regression with adjusted standard errors accounting for matching and the fact that 103 non-donors were matched twice. For each regression analysis we tested for differences between donors and non-donors while also correcting for the covariates age, gender, start year (date of donation or enrollment in population-based study), education level, pre-existing hypertension, baseline serum creatinine, and baseline eGFR, as well as weight, height, alcohol use, and smoking status at follow-up. To evaluate potential effect modification, interactions were tested by age, gender, and follow-up duration in the final multivariate model of creatinine and eGFR. The results were not corrected for multiple testing.

## Results

### Characteristics of the study participants

**Fig. 1:**
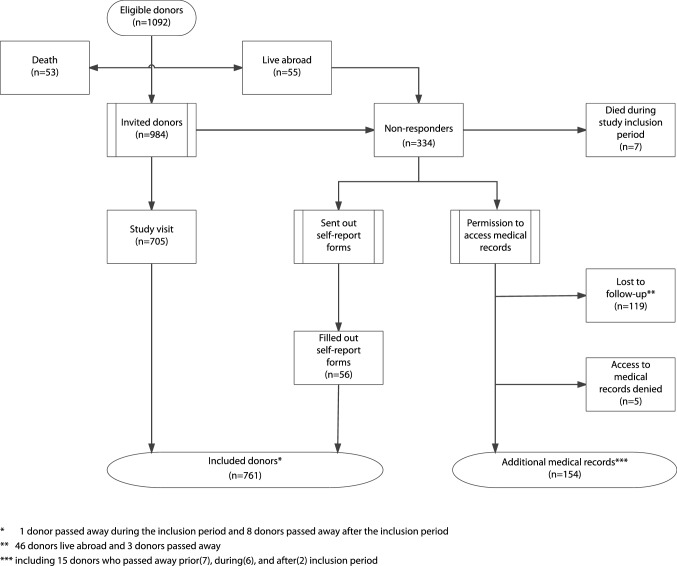
Flowchart of inclusion in the live kidney donor process

Between 1981 and 2011, a total of 1092 live kidney donations were performed at our center and all donors were eligible for inclusion. A total of 761 donors were matched with non-donors, including 705 who visited the outpatient clinic (Fig. 1). The donors comprised 429 living-related (54.1%) and 332 living-unrelated (45.9%) live kidney donations. The median follow-up time for donors was 8.0 (5.1–11.9) years. Non-donors from the Rotterdam Study and SHIP were included; a total of 11,251 individuals participated in these population-based cohort studies. After applying our exclusion criteria, 9270 non-donors were eligible for 2:1 matching, resulting in 1522 non-donors being included in the study (54% from the Rotterdam Study and 46% from SHIP). The median follow-up time for non-donors was 7.0 (5.4–10.9) years. Age, baseline systolic blood pressure, ethnicity, and education were significantly different between donors and non-donors (Table 1).

**Table 1:** Baseline characteristics of included live kidney donors at the time of donation and matched nondonors at the time of enrolment in population based studies; reported p-values are not adjusted for matching

Characteristics	Donors (n = 761)	Matched nondonors (n = 1522)	*p* value
	Median (25–75p)/N (%)	Median (25–75p)/N (%)	
Age (years)	51.9 (42.8–60.1)	51.0 (37.8–57.9)	*p* < *0.001*
Gender (male)	318 (41.8)	636 (41.8)	*p* = 1.000
Ethnicity (white)	681 (89.5)	1434 (94.2)	*p* = *0.001*
BMI (kg/m^2^)	25.9 (23.4–28.4)	25.5 (22.9–28.2)	*p* = 0.176
Systolic blood pressure (mmHg)	130.0 (120.0–140.0)	125.5 (114.0–142.0)	*p* = *0.010*
Diastolic blood pressure (mmHg)	79.0 (70.0–85.0)	77.0 (70.0–85.0)	*p* = 0.964
eGFR (ml/min/1.73 m^2^)	92.6 (79.8–103.8)	90.0 (78.9–102.3)	*p* = 0.160
Serum glucose (mmol/l)	4.9 (4.3–5.4)	5.0 (4.7–5.4)	*p* = 0.778
Pre-existing hypertension^a^	299 (39.3)	592 (38.9)	*p* = 0.891
Pre-existing cardiovascular disease	13 (1.7)	28 (1.8)	*p* = 0.956
Smoking	400 (52.6)	867 (57.0)	*p* = 0.051
Alcohol use			*p* = 0.560
Never/rare	340 (44.7)	703 (46.2)	
≤7 glasses/week	294 (38.6)	553 (36.3)	
>7 glasses/week	127 (16.7)	266 (17.5)	
Education degree			*p* = *0.011*
Primary	86 (11.3)	159 (10.4)	
Secondary	418 (54.9)	782 (51.4)	
Tertiary	257 (33.8)	581 (38.2)	

^a^Defined as use of antihypertensive medication, systolic blood pressure ≥ 140 mmHg or diastolic blood pressure ≥ 90 mmHg

### Effect of donation on kidney function

**Fig. 2:**
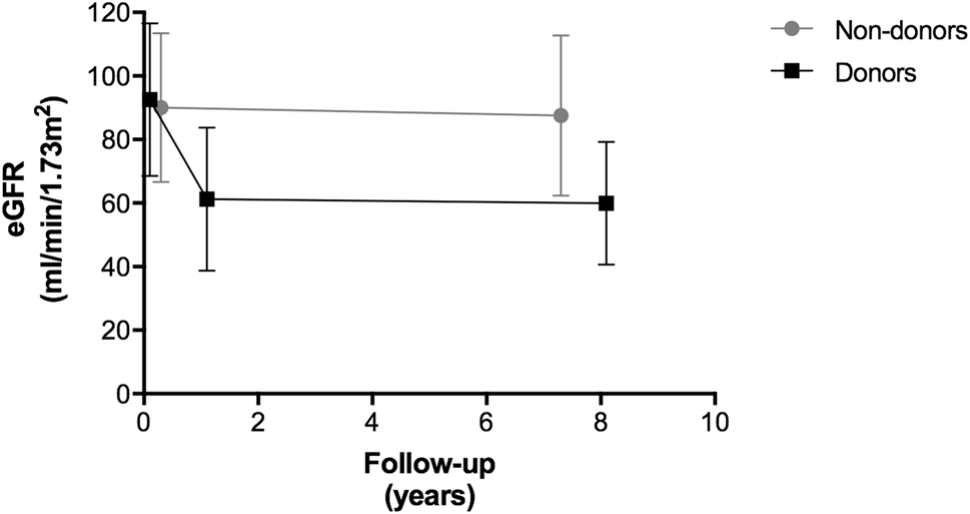
Overall eGFR levels over time: baseline and 7 years for non-donors, and baseline, 1 year, and 8 years for donors

In the donor population, baseline eGFR was 92.6 ml/min/1.73 m^2^ (IQR 79.8–103.8 ml/min/1.73 m2), one-year eGFR was 59.0 ml/min/1.73 m^2^ (IQR 50.5–68.6 ml/min/1.73 m^2^), and median eGFR at follow-up after 8 years 59.9 ml/min/1.73 m^2^ (IQR 51.4–70.7 ml/min/1.73 m^2^) (Fig. 2). The serum creatinine levels for donors were 77.0 μmol/l (IQR 68.0–87.0 μmol/l) at baseline, 106.0 μmol/l (IQR 93.0–121.0 μmol/l) at one-year and 100.0 μmol/l (IQR 87.5–114.0) μmol/l at 8-year follow-up. Sixteen donors (2.4%) and 10 non-donors (1.2%) developed microalbuminuria (*p* = 0.09). These donors had a median eGFR of 57.9 ml/min/1.73 m^2^ (IQR 47.7–68.2 ml/min/1.73 m2) and median serum creatinine of 99.5 μmol/l (IQR 86.1–112.9 μmol/l) at follow-up. Two donors (0.3%) developed ESRD.

### Primary outcomes

In donors, serum creatinine was significantly higher (+26.03 μmol/l (95% CI 24.17; 27.89)) and the eGFR significantly lower (− 27.23 ml/min/1.73 m2 (95% CI − 28.61; − 25.85)) compared to non-donors. The eGFR declined 31.7 percent (95% CI 29.9; 33.5) more among donors than non-donors (all *p* < 0.001, Table 2). The incidence of ESRD was not significantly different between donors and non-donors (OR 0.56, 95% CI 0.19; 1.71).

**Table 2:** Long-term outcomes of live kidney donors compared with matched nondonors after an overall median follow-up of 7.3 years

Outcome	Effect estimate^a^	95% CI	*p* value
Primary outcomes			
Change in serum creatinine (μmol/l)	26.03	24.17; 27.89	*p* < *0.001*
Change in eGFR (ml/min/1.73 m^2^)	− 27.23	− 28.61; − 25.85	*p* < *0.001*
eGFR decline (%)	− 31.70	− 33.46; − 29.94	*p* < *0.001*
ESRD	0.56	0.19; 1.71	*p* = 0.311
Secondary outcomes			
BMI (kg/m^2^)	0.02	− 0.04; 0.07	*p* = 0.583
New-onset diabetes	1.14	0.71; 1.84	*p* = 0.585
New-onset hypertension^b^	0.45	0.33; 0.62	*p* < *0.001*
Cardiovascular event	1.06	0.64; 1.74	*p* = 0.823
Cardiovascular mortality	0.13	0.01; 1.24	*p* = 0.077
Overall mortality	0.06	0.05; 0.08	*p* < *0.001*
SF-12 physical component score	− 1.36	− 2.38; − 0.33	*p* = *0.010*
SF-12 mental component score	− 4.61	− 5.75; − 3.47	*p* < *0.001*
EQ-5D	0.06	0.05; 0.08	*p* < *0.001*

^a^β for continuous outcome, odds ratio for dichotomous outcome

^b^Study participants with pre-existent hypertension were excluded from analysis. Defined as use of antihypertensive medication, systolic blood pressure ≥ 140 mmHg or diastolic blood pressure ≥ 90 mmHg. Model adjusted for: age, gender, start year (date of donation or enrolment in population-based study), education degree level, pre-existing hypertension, baseline serum creatinine, baseline eGFR, and weight, height, alcohol use, and smoking status at follow-up. The number of donors for the outcomes varied between 747 and 756, and for non-donors between 605 and 960

### Secondary outcomes

The crude incidence for secondary outcomes among donors was 5.2% for new-onset diabetes, 39.2% for new-onset hypertension, 3.9% for cardiovascular events, 0.1% cardiovascular mortality, and 1.2% for overall mortality.

Secondary outcomes showed no significant differences between donors and non-donors for BMI (0.02, (95% CI − 0.04; 0.07), incidence of diabetes (OR 1.14, 95% CI 0.71; 1.84), cardiovascular events (OR 1.06, 95% CI 0.64; 1.74), and cardiovascular mortality (OR 0.13, 95% CI 0.01; 1.24). A significantly lower risk of developing new-onset hypertension (OR 0.45, 95% CI 0.33; 0.62) and overall mortality (OR 0.06, 95% CI 0.05; 0.08) was found among donors. Of the entire 1981–2010 donor population (n = 1092), 80 donors died, including 38 due to cancer and 15 due to a cardiovascular event.

### Quality of life

The health-related quality of life score was significantly higher among donors with 0.06 higher score for EQ-5D (95% CI 0.05; 0.08 on a scale of − 0.59 to 1.00). The SF-12 physical and mental component scores were both significantly lower among donors: − 1.36 (95% CI − 2.38; − 0.33) and − 4.61 (95% CI − 5.75; − 3.47), respectively, on a scale of 0–100.

## Discussion

In this European study of live kidney donors and propensity score matched non-donors from the general population, we demonstrated that the long-term prognosis of people that underwent a live kidney donation is identical to non-donors. Moreover, the lower eGFR after donation due to reduction of parenchymal kidney volume did not result in a higher incidence of ESRD, cardiovascular disease or mortality compared to non-donors. This study adds to the current literature, because it suggests that recently reported negative outcomes of kidney donation may partially be explained by the selection of the non-donor control group. This confirms our recent methodological assessment of previous studies analyzing long-term safety of live kidney donation [19].

As expected, eGFR among donors was decreased at follow-up as compared to the general population (Table 2). The decrease in eGFR is likely attributable to the donation procedure, since the eGFR declined in the first year after donation and remained stable thereafter. Thus, this eGFR-loss likely reflects the loss of nephron mass after donation. However, eGFR in donors should be interpreted carefully and may deviate substantially from measured GFR. For example, Tan et al. compared CKD-EPI GFR with measured GFR using iothalamate in 64 kidney donors [37]. They showed that more than half of kidney donors had eGFR < 60 ml/min/1.73 m^2^, whereas only 25% had a measured GFR below this level. This misclassification mainly affected donors > 55 years. This implies that the true GFR in our cohort of kidney donors may be better than estimated by the CKD-EPI equation.

In a recent published systematic review and meta-analysis comparing mid- and long-term health risks in living kidney donors and matched non-donors [38] the outcomes for eGFR, creatinine, all-cause mortality, cardiovascular events, the incidence of type 2 diabetes, and health-related quality of life as measured with the Short-Form 12 among donors were similar to our findings. As opposed to their results, our study had a lower risk of hypertension and no increased risk for ESRD. This may be explained by our annual outpatient follow-up appointments in which blood pressure and blood- and urine analysis are performed and abnormalities could be detected early. Although we found a statistically significant difference in the SF-12 physical and mental component scores, the difference between donors and non-donors was < 5-points which is not deemed clinically relevant [29]. In contrast, a higher EuroQoL score was seen among donors. This can be explained since a previous study has found that when indicating a health problem on the EQ-5D a significantly lower mean SF-12 component scores for all dimensions is found [39]. Therefore, these at first sight contrasting results should not be seen as a contradiction.

Several studies on live kidney donation have used a similar comparison design with matched non-donors and reported similar outcomes in overall mortality and cardiovascular events [4, 8, 10]. However, others have reported contradicting results [15, 40]. This may be explained by differences in study design, questioning the comparability of donors and non-donors and the reliability of the results [19]. Live kidney donors are healthy individuals and submitting them to a surgical procedure stretches the Hippocratic oath taken by physicians. Therefore, prior to donation, a well-balanced decision has to be taken regarding the suitability and the risk estimation for donation. By extensively screening donors the healthiest individuals are selected, which may limit the comparability with a general selection of non-donors. Comparing donors to a general selection of the background population can underestimate risks added by the donation [9]. Matching donors to selective non-donors with similar baseline health status, as was done in this study, can overcome this limitation. We have added these factors to our study design by matching and adjusting for multiple health characteristics. Thus, this study aimed to include representative non-donors in Western populations. Although at first sight age, ethnicity and systolic blood pressure were statistically different between donors and non-donors, a closer look at the actual difference are thought to be negligible when advising potential donors by transplant professionals. The next step would be to predict the attributable risk for individual donors prior to donation [17].

A strength of our study is that, in addition to national registry data as used in other studies [15, 16], we collected individual donor and non-donor data on medical conditions, physical examinations, laboratory tests, medication use, which were all manually verified in medical records and had minimal missing data (< 8%). Second, compared to previous studies [4, 9, 10, 15, 16, 18, 19] a strength of our study lies in the availability of repeated measurements of serum creatinine and albuminuria. Furthermore, compared to previous studies the current study design was optimized for donor and non-donor comparison. First, we matched on more baseline health characteristics than previous studies, including eGFR and comorbidity. Second, we adjusted for current lifestyle factors such as alcohol use and smoking. Finally, we performed propensity score matching which is suitable for observational data to balance both groups on a large number of covariates without losing a large number of observations,

Our study has a number of limitations. First, data on the type of antihypertensive medication was not available from our data sources for non-donors. Second, blood pressure consisted of a single measurement in donors but on average 2–3 measurements in non-donors. Third, for SHIP participants, percutaneous coronary intervention and coronary artery bypass surgery were not defined as cardiovascular events. Fourth, EQ-5D scores were only available in the Rotterdam Study and SF-12 scores were only available in the SHIP. Finally, serum creatinine and eGFR are only estimates of kidney function that may deviate substantially from measured GFR in donors [37].

In conclusion, live kidney donors have reduced eGFR compared to non-donors, occurring in the first year after donation and stabilizing thereafter for at least 8 years with similar outcomes in terms of morbidity and mortality as non-donors. A lower risk of hypertension was found and no difference was found in cardiovascular risk. However, kidney function in donors may be further compromised with unforeseen circumstances. With the knowledge of this risk, albeit small, donors should be well-informed by the medical team before donation and be offered lifelong follow-up thereafter. By monitoring kidney function, donors at risk may be identified at an early stage and adequate treatment may be offered. We consider this approach a prerequisite to legitimize a living kidney donor program.

## Electronic supplementary material

Below is the link to the electronic supplementary material.

Supplement 1. Supplemental Table S1. STROBE Statement—Checklist of items that should be included in reports of cohort studies. Supplement 2. Supplemental Methods. Supplement 3. Supplemental Figure S1. Number of donations per year (1981-2010) (DOCX 730 kb)
