# Supplementary material for: Long-term prognosis after kidney donation: a propensity score matched comparison of living donors and non-donors from two population cohorts
Source: Eur J Epidemiol. 2020 May 21;35(7):699–707. doi: 10.1007/s10654-020-00647-y (PMC7387377; doi:10.1007/s10654-020-00647-y)

**Supplemental Table S1. STROBE Statement—Checklist of items that should be included in reports of cohort studies**

|  | Item no. | Recommendation | Page |
| --- | --- | --- | --- |
| **Title and abstract** | 1 | (*a*) Indicate the study’s design with a commonly used term in the title or the abstract. | 1 |
|  |  | (*b*) Provide an informative and balanced summary of what was done and what was found. | 3 |
| Introduction | | |  |
| Background/rationale | 2 | Explain the scientific background and rationale for the investigation being reported. | 4 |
| Objectives | 3 | State specific objectives, including any pre-specified hypotheses. | 4 |
| Methods | | |  |
| Study design | 4 | Present key elements of study design early in the paper. | 5 |
| Setting | 5 | Describe the setting, locations, and relevant dates, including periods of recruitment, exposure, follow-up, and data collection. | 5 |
| Participants | 6 | (*a*) Give the eligibility criteria and the sources and methods of selecting participants. Describe follow-up methods. | 6 |
|  |  | (*b*) For matched studies, give matching criteria and number of exposed and unexposed. | 7 |
| Variables | 7 | Clearly define all outcomes, exposures, predictors, potential confounders, and effect modifiers. Give diagnostic criteria if applicable. | 6-7 |
| Data sources/ measurement | 8* | For each variable of interest, give sources of data and details of the methods of assessment (measurement). Describe the comparability of assessment methods if there is more than one group. | 5 |
| Bias | 9 | Describe any efforts to address potential sources of bias. | 7 |
| Study size | 10 | Explain how the study size was arrived at. | 6 |
| Quantitative variables | 11 | Explain how quantitative variables were handled in the analyses. If applicable, describe which groupings were chosen and why. | 7 |
| Statistical methods | 12 | (*a*) Describe all statistical methods, including those used to control for confounding. | 8 |
|  |  | (*b*) Describe any methods used to examine subgroups and interactions. | 8 |
|  |  | (*c*) Explain how missing data were addressed. | 7 |
|  |  | (*d*) If applicable, explain how loss to follow-up was addressed. |  |
|  |  | (*e*) Describe any sensitivity analyses. | 8 |
| Results | | |  |
| Participants | 13* | (a) Report number of individuals at each stage of the study: e.g., potentially eligible, examined for eligibility, confirmed eligible, included in the study, completing follow-up, and analyzed. | 9 |
|  |  | (b) Give reasons for non-participation at each stage. | 9 |
|  |  | (c) Consider use of a flow diagram. | Fig 1 |
| Descriptive data | 14* | (a) Give characteristics of study participants (e.g., demographic, clinical, social) and information on exposure and potential confounders. | Table1 |
|  |  | (b) Indicate the number of participants with missing data for each variable of interest. | Table1 |
|  |  | (c) Summarize follow-up time (e.g., average and total amount). | 9 |
| Outcome data | 15* | Report number of outcome events or summary measures over time. | 9-10 |
| Main results | 16 | (*a*) Give unadjusted estimates and, if applicable, confounder-adjusted estimates and their precision (e.g., 95% confidence interval). Clearly indicate which confounders were adjusted for and why they were included. | 10 |
|  |  | (*b*) Report category boundaries when continuous variables were categorized. | 10 |
|  |  | (*c*) If relevant, consider translating estimates of relative risk into absolute risk for a meaningful time period. |  |
| Other analyses | 17 | Report other analyses done: e.g., analyses of subgroups and interactions, and sensitivity analyses. | Suppl |
| Discussion | | |  |
| Key results | 18 | Summarize key results with reference to study objectives. | 11 |
| Limitations | 19 | Discuss study limitations taking into account sources of potential bias or imprecision. Discuss both the direction and magnitude of any potential bias. | 13 |
| Interpretation | 20 | Give a cautious overall interpretation of results considering objectives, limitations, multiplicity of analyses, results from similar studies, and other relevant evidence. | 11 |
| Generalizability | 21 | Discuss the generalizability (external validity) of the study results. | 12 |
| Other information | | |  |
| Funding | 22 | Give the source of funding and the role of the funders for the present study and, if applicable, for the original study on which the present article is based. | 14 |

**Supplemental Methods**

**Restriction, multiple imputation, and matching**

A total of 11,251 participants from the SHIP (n=4308) and the Rotterdam Study (n=6943) were eligible as non-donors. After restricting participants (total n=1981; SHIP n= 816, Rotterdam Study n=1165) with pre-existing diabetes, an (e)GFR < 60 ml/min/1.73 m^2^, and BMI > 40 kg/m^2^, a total of 9270 participants were identified (SHIP n= 3492, Rotterdam Study n= 5778).

Due to missing baseline data on BMI (5.9%), ethnicity (2.7%), eGFR (7.8%), systolic (5.5%) and diastolic (5.5%) blood pressure, pre-existing hypertension (4.6%), pre-existing cardiovascular events (0.2%), serum glucose level (7.5%), current smoking (0.5%), alcohol use (3.5%), and education level (2.6%), multiple imputations based on the method of chained equations[^32^](#_ENREF_32) was performed for 1092 donors, 3492 participants from SHIP, and 5778 participants from the Rotterdam Study.

All 761 donors included in the study were matched based on propensity scores to two non-donors from among the 9270 participants with replacement. Matching covariates were age, gender, year of donation/enrollment in the population-based cohort study, BMI, ethnicity, eGFR, systolic and diastolic blood pressure, pre-existing hypertension, pre-existing cardiovascular events, serum glucose level, current smoking, alcohol use, and education level. Matching was performed using the optimal matching algorithm implemented in the R package as optmatch.[^34^](#_ENREF_34) This algorithm finds the best match based on all variables together. On average (over the 20 multiple imputed data sets), 103 non-donors were used more than once to match with a donor.

**Supplemental Figure S1. Number of donations per year (1981-2010)**


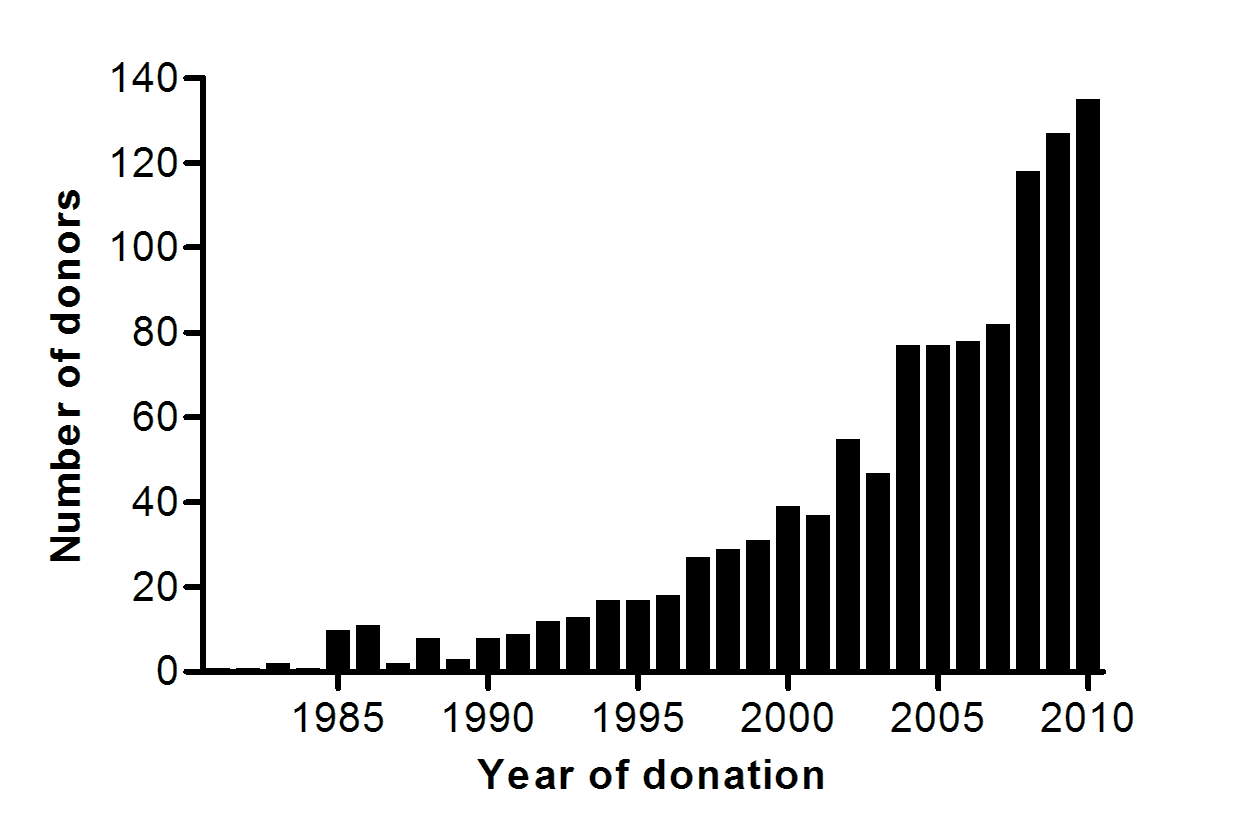

Supplement: Supplementary file 1 — Supplement 1. Supplemental Table S1. STROBE Statement—Checklist of items that should be included in reports of cohort studies. Supplement 2. Supplemental Methods. Supplement 3. Supplemental Figure S1. Number of donations per year (1981-2010) (DOCX 730 kb) [file 10654_2020_647_MOESM1_ESM.docx]
